# Point-of-contact Interactive Record Linkage (PIRL): A software tool to prospectively link demographic surveillance and health facility data

**DOI:** 10.12688/gatesopenres.12751.2

**Published:** 2018-01-11

**Authors:** Christopher T. Rentsch, Chodziwadziwa Whiteson Kabudula, Jason Catlett, David Beckles, Richard Machemba, Baltazar Mtenga, Nkosinathi Masilela, Denna Michael, Redempta Natalis, Mark Urassa, Jim Todd, Basia Zaba, Georges Reniers

**Affiliations:** 1 Department of Population Health, London School of Hygiene & Tropical Medicine, London, WC1E 7HT, UK; 2 MRC/Wits Rural Public Health and Health Transitions Research Unit (Agincourt), School of Public Health, Faculty of Health Sciences, University of the Witwatersrand, Johannesburg , 2193, South Africa; 3 SELECT Star, Atlanta, GA, 30309, USA; 4 Independent Researcher, London, UK; 5 The Tazama Project, National Institute for Medical Research, Mwanza, Tanzania; 6 District Medical Officer, Ministry of Health Tanzania, Magu District, Tanzania

**Keywords:** data linkage, interactive record linkage, health and demographic surveillance systems, health facility, sub-Saharan Africa

## Abstract

Linking a health and demographic surveillance system (HDSS) to data from a health facility that serves the HDSS population generates a research infrastructure for directly observed data on access to and utilization of health facility services. Many HDSS sites, however, are in areas that lack unique national identifiers or suffer from data quality issues, such as incomplete records, spelling errors, and name and residence changes, all of which complicate record linkage approaches when applied retrospectively. We developed Point-of-contact Interactive Record Linkage (PIRL) software that is used to prospectively link health records from a local health facility to an HDSS in rural Tanzania. This prospective approach to record linkage is carried out in the presence of the individual whose records are being linked, which has the advantage that any uncertainty surrounding their identity can be resolved during a brief interaction, whereby extraneous information (e.g., household membership) can be referred to as an additional criterion to adjudicate between multiple potential matches. Our software uses a probabilistic record linkage algorithm based on the Fellegi-Sunter model to search and rank potential matches in the HDSS data source. Key advantages of this software are its ability to perform multiple searches for the same individual and save patient-specific notes that are retrieved during subsequent clinic visits. A search on the HDSS database (n=110,000) takes less than 15 seconds to complete. Excluding time spent obtaining written consent, the median duration of time we spend with each patient is six minutes. In this setting, a purely automated retrospective approach to record linkage would have only correctly identified about half of the true matches and resulted in high linkage errors; therefore highlighting immediate benefit of conducting interactive record linkage using the PIRL software.

## Introduction

The amount of collected data is ever-increasing in various sectors, including healthcare and government administration. While each individual data source holds value and was likely created for a specific purpose, researchers could study more complex relationships by combining data sources holding information on the same entity or individual. A recent Wellcome Trust report detailed how record linkage – the matching of an individual’s records between two or more data sources – adds to the value of medical research in low- and middle-income as well as high-income countries ^1^. Broadly, record linkage can increase the range of questions that could be asked, provide a historical perspective necessary for some studies, improve the statistical properties of analyses, and make better use of resources.

The statistical framework for record linkage was largely developed in the 1950s ^2^ and 1960s ^3^. Two popular methods of record linkage have been used to combine data sources. Deterministic record linkage ^4^ is a rule-based approach that typically requires exact matching on a set of identifiers existing in all data sources. Probabilistic methods ^5^,^7^ can be employed to assign weights based on the (dis)similarity of identifiers (e.g., name, sex, and date of birth) between records.

In the United Kingdom, researchers use record linkage to merge the Clinical Practice Research Datalink – one of the largest databases of longitudinal medical records from primary care in the world – to a variety of other existing data sources that hold data on cardiovascular and cancer events, hospitalisation, and mortality ^8^. Publications using this data infrastructure cover a vast range of topics, including studies showing the absence of an association between measles, mumps, and rubella (MMR) vaccine and autism ^9^, cardiovascular risk after acute infection ^10^, and the association between body mass index and cancer ^11^.

Located in several low- and middle-income countries, health and demographic surveillance systems (HDSS) are effective and comprehensive data collection systems that primarily measure the fertility, mortality, and other self-reported health information of an entire population. However, such self-reports usually lack detail and accuracy about the clinical events and services received, and their retrospective nature means they quickly become dated. Linking an HDSS database to data from a health facility that serves the HDSS population produces a research infrastructure for generating directly observed data on access to and utilization of health facility services ^12^.

Many HDSS sites, contrary to record linkage studies conducted in high-income countries, are in areas that lack unique national identifiers or suffer from data quality issues, such as incomplete records, spelling errors, and name and residence changes, all of which complicate both deterministic and probabilistic approaches when applied retrospectively. In these settings, a semi-automatic record linkage process that incorporates manual inspection of potential matches, such as interactive record linkage ^13^,^14^, is preferred. In our implementation of interactive record linkage, which we call point-of-contact interactive record linkage (PIRL), we carry out the manual inspection of potential matches identified by our linkage algorithm in the presence of the individual whose records are being linked. This prospective approach to record linkage has the advantage that any uncertainty surrounding their identity can be resolved during a brief interview, whereby extraneous information (e.g. household membership) can be referred to as an additional criterion to adjudicate between multiple potential matches. It also provides an opportunity to authenticate individuals who can legitimately be linked to more than one record in the HDSS because they have resided in more than one household. Finally, ethical and privacy concerns are properly addressed with PIRL as it offers an advantage to seek informed consent and individuals are made fully aware of how their data are being used.

There are numerous publicly and commercially available record linkage software packages. Herzog *et al.* ^15^ adapted a comprehensive checklist ^16^ for evaluating record linkage software, including questions regarding the amount of control the user has over the record linkage methodology, data management and standardisation, and post-linkage functions. Many of the available software packages are designed for batch linkages, such as those used in purely automated retrospective linkage ^17^,^18^. Given the novelty of the PIRL approach where searches are individually supervised, we opted to build our own software package to suit our specific needs. By designing our own software, we maintained full control over the specification of the linkage algorithm, including the match parameters, weights, agreement rules, string comparators, and how to handle missing data. We also required the ability to save session-specific notes that can be retrieved in future linkage sessions.

We introduced our PIRL software to prospectively link health records to HDSS records in a rural ward in northeast Tanzania. An analysis of the data created by our implementation of the software and how it compares to purely automated retrospective linkage has previously been published ^19^. This paper describes our implementation of this software, and we attach a GitHub link ^20^ to the full source code for others to download and amend to their own research needs.

## Methods

### Data sources

The Kisesa observational HIV cohort study was established in 1994 and is located in a rural ward in the Magu district of Mwanza region in northwest Tanzania. It comprises demographic surveillance carried out through household interviews and population-based HIV surveillance based on individual serological tests and interviews. The HDSS databases include biannual rounds (31 to date) of household-based surveys that collect information on births, pregnancies, deaths, in- and out-migration, and spousal and parent-child relationships. One major weakness of the Kisesa HDSS is the lack of reconciling records of individuals who move households within the HDSS area. Therefore, while an HDSS ID is unique to a single individual, some individuals may have multiple HDSS IDs if they resided in more than one household in the HDSS area since the start of the HDSS in 1994. There have been eight rounds of HIV surveillance conducted every three years, with a detailed questionnaire on sexual behaviour and partnership factors, fertility outcomes, HIV-related knowledge, and use of health services. Individuals who participate in an HIV surveillance round are given a unique identifier, and their current unique identifier from the HDSS is also cross-referenced on their record.

A government-run health centre is situated in the Kisesa HDSS catchment area. Three clinics located in the Kisesa Health Centre were initially targeted as record linkage sites: the HIV care and treatment centre (CTC), the HIV testing and counselling clinic (HTC), and the antenatal clinic (ANC) which includes prevention of mother-to-child transmission services; all of which operate according to national guidelines and protocols. The CTC databases have been fully digitised, and data clerks regularly update and run data checks on these data. For the ANC and HTC clinics, we developed electronic data capture systems and digitised the paper-based logbooks.

### Implementation

Our computer software utilises a probabilistic search algorithm to identify and rank potential matches in the HDSS database (n=110,000). The algorithm incorporates the following parameters or data fields: up to three names for the individual; sex; year, month, and day of birth; village and sub-village; up to three names of a household member; and up to three names for the ten-cell leader of the patient. A ten-cell leader is an individual who acts as a leader for a group of ten households and these positions have been relatively stable over time. The algorithm used for searching possible matches and ranking them is based on the Fellegi-Sunter record linkage model ^2^,^3^, with match probabilities ( *m _i_*) that have been adopted from a pilot study in the Agincourt HDSS ^21^. The *u*_*i*_ probabilities, defined as chance agreement between two records which are true non-matches, were derived from the Kisesa HDSS data consistent with previous literature7. Let *M* be a set of true matches and *U* be a set of true non-matched record pairs. Two individual agreement probabilities are defined for each field *i* in record pair *j* as follows:

match probability: *m _i_* = P(field *i* agrees | *j* ϵ *M*)                          (1.1)
  unmatch probability: *u _i_* = P(field *i* agrees | *j* ϵ *U*)                          (1.2)

For a given field with match probability *m _i_* and unmatch probability *u _i_*, the software calculates the matching weights *w _ai_* as = log _2_[ *m _i_*/ *u _i_*] for fields where both datasets agree, and *w _di_* as = log _2_[(1- *m _i_*)/(1- *u _i_*)] where they disagree. Assuming independence of observations across the fields, the match score is computed by summing the weights across all fields ^3^,^15^.

Agreement conditions vary for each of the parameters. Spelling errors, the use of more than one name (including nicknames), and interchangeable name order complicate locating an exact match between names in these databases; thus, the linkage algorithm allows for all pairwise comparisons between reported names and names found in the HDSS. In addition, the software uses a Jaro-Winkler string comparator approach to compare the name fields between the two data sources ^22^. Previous research has shown the Jaro-Winkler method produces similar results to Double Metaphone and Soundex string comparators in a southern African context ^21^. A Jaro-Winkler score ≥0.8 was considered a match for each collected name. Sex, village, and sub-village required an exact match, while the year of birth could differ by up to two years.

### Operation

A full user guide including screen shots and step-by-step instructions on how we operationalise this software is attached ( Supplementary File 1). Briefly, as individuals arrive to any of the target clinics, a fieldworker introduces him/herself and then invites the attendee to take part in the linkage study, which involved a brief interview. The primary goals of the brief interview are to explain the study, seek informed consent, and identify the HDSS records of all participants with a residency history in the HDSS.

Our team uses a dedicated desk located within the clinic, but out of the way of normal clinic operations, to conduct the brief interviews, and therefore did not interrupt or interfere with clinical practice. While we highly recommend ensuring privacy during each patient interaction, the interview only involves asking for demographic information, such as name, sex, birthdate, and residence details, and does not ask for any medical information. In addition, all collected data from a previous session is cleared from the system at the end of each patient interaction. Therefore, to enhance the accuracy of the data, we allow patients to watch their information be entered into the software and ask them to verify what has been collected.

**Figure 1.**
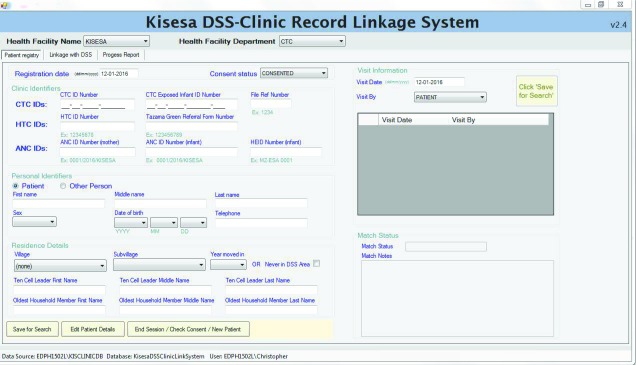
User interface of Point-of-contact Interactive Record Linkage (PIRL) software.

The first step after obtaining written consent is to collect all clinic identifiers for the patient. The software uses these clinic identifiers to retrieve previously collected information and matches made on patients interviewed during a prior visit. After all clinic identifiers are collected, personal and residence details are entered into the system ( Figure 1). Information from most of these fields contribute to the linkage algorithm described in the Implementation section above.

Once all personal and residence details are entered, the user initiates an initial search through the HDSS data source. The software computes a match score for each record in the HDSS database, ranks them from highest to lowest based on match score, and outputs the top 20 records within 15 seconds. While manually searching through these potential matches, the user can view the full list of household members associated with each HDSS record. The user can then inquire with the patient to identify which HDSS record(s), if any, are a true match.

An important feature of this software is the ability to perform multiple search attempts for a single patient. If an initial search attempt does not result in a match, the user can further inquire into the possible use of nicknames, maiden names, or residency episodes at other addresses, and perform consecutive searches with this updated information. If one or more HDSS records are not found, the user can enter details of the missing records into a free-text field called “match notes.” These match notes are retrieved by clinic identifiers and can be used to guide interviews and searches during subsequent visits. When a clinic identifier is entered into the system that has already been collected, the software automatically displays the match status (e.g., matched, not matched) and saved matched notes to the user. The dates of all follow-up visits are automatically logged into the system.

Because we use this software in an area without reliable internet connectivity, we perform manual backups and syncs of the back-end data at the end of each working day as a way to mitigate any risk for loss of collected data. Full details on the import and export routines can be found in Annex 2 of the attached user guide ( Supplementary File 1). Briefly, the data manager exports a backup file from each of the user’s machines using SQL Server Management Studio (SSMS). Then, the backup files are imported into SSMS on the data manager’s machine, and a SQL program automatically merges, updates, and collates the data collected from previous days. Finally, the data manager exports the combined backup file and imports it onto each of the user machines. Source code for these import and export routines can also be found on GitHub.

We employ data integrity checks within the software and on the back-end data. Due to the importance of clinical identifiers, all ID fields require double entry. Furthermore, HTC IDs are ensured through modulo-97 check digits, and ANC and CTC IDs have specific formats that the software confirms. The software also displays warning messages to the user if they attempt to match to a record that has an absolute difference in birth year of >10 years or the sum of the Jaro-Winkler name scores is ≤1.6.

To validate the matches in the back-end database, the lead author performs periodic and manual, back-end inspection of the data. These data integrity checks flag individuals who are matched to multiple HDSS records with large age differences (>10 years), of conflicting sex, within the same household, or with overlapping residency episodes in which one record’s start date occurred before another record’s end date. Over 18 months, only eight (0.2%) out of 3,456 matches were deemed unlikely and were deleted from the back-end database.

### System requirements

The user interface (UI) portion of the software was coded using C# language in Microsoft Visual Studio 2013 Community edition. The database management system was coded in Microsoft SQL Server 2012 Express. The software has been developed for machines running a Windows 7 operating system.

Users who wish to edit source code to tailor the software to their specific needs will need both Visual Studio and SSMS. However, users who only need to run the software will need SSMS alone. 

Full installation instructions can be found in Annex 1 of the attached user guide ( Supplementary File 1).

## Use cases

### Input dataset

Due to the nature of the software and its requirement for personally identifiable information, we are unable to provide real HDSS data used in our implementation of the software. However, we did create a dataset of 100 fake HDSS records that randomly sampled information found in the real data. Each field was sampled separately to break any links of information that could identify an individual. Spelling alterations, change of names, and other minor errors to birthdays or residence details were made to make the example cases described below more realistic to what we experience in the field. The data and a codebook for the fake input dataset are attached ( Supplementary File 2). The script used to create the fake input dataset is also attached ( Supplementary File 3).

### Output datasets

The software creates four password-encrypted tables and stores them in SSMS. The first table, called the ‘Registry’, stores clinic identifiers, personal and residence details reported by the patient and entered by the fieldworker into the main view of the software ( Figure 1). A new record is created for each search attempt. The second table, called ‘Matches’, stores all matches made to HDSS records, including the HDSS identifier, match score, and the rank of the match. The third table, called ‘Notes’, holds the collection of match notes made during an interview. The fourth table, called ‘Visits’, is a file containing all visit dates for each patient.

Three auto-generated identifiers are used to link records that pertain to a specific individual between the four back-end data tables: the local machine name, a session ID, and a record number. For each local machine, a session ID consisting of numerical values for year, month, day, hour, minute, and second gets automatically created at the beginning of a new session (e.g., ‘20170601093000’ for a session initiated at exactly 9:30:00am local time on 1 June 2017). Within each session, a six-digit record number is created and iterates for each search attempt within a session. Whenever a match is made (table 2), match notes are stored (table 3), or a visit date is recorded (table 4), the values for the machine name, session ID, and record number are stamped on those records.

An example output database from the cases below and its codebook are attached ( Supplementary File 4).

### Case 1

The patient enters the CTC and agrees to take part in this study. The fieldworker collects his CTC ID and enters it into the system along with the personal and residence details he reports ( Table 1). The software displays the top 20 potential matches to the fieldworker. The fieldworker selects the top ranked record to view the entire household membership and confirms the reported co-resident is listed. There are minor spelling errors in the names, but the year of birth, years of residency, and residence details match exactly. Thus, the fieldworker assigns the match to this record and ends the search as all reported residency episodes were found. The fieldworker saves a match note that says, “All reported residency episodes found.” The fieldworker then stores the visit date and thanks the patient for his time.

**Table 1.** Personal identifiers used for three case patients sampled from the fake dataset with varying numbers of residency episodes.

	Case 1	Case 2	Case 3		
Residency episode	1	1	1	2	3
**Clinic ID(s)**	CTC: 77-10-4545- 253004	ANC: 1234/2017/KISESA	HTC: 44618061		
		HTC: 44447050			
**First name**	PETER	PASTORY	SUZANNE	SUZANNE	SUZANNE
**Second name**	JAKKU	SWAKALA	LENARD	JONAS	JONAS
**Third name**		TIMOS	WILLIAMS	ZABRON	ZABRON
**Sex**	M	F	F	F	F
**Year of birth**	2004	1984	1980	1980	1980
**Month of birth**	8	9			
**Day of birth**	15				
**Village**	KANYAMA	KANYAMA	KISESA	Outside HDSS area	IHAYABUYAGA
**Subvillage**	CHANGABE	NYAN’HELELA	KISESA KATI		ILENDEJA
**Residence start** **year**	2012	2010	1995	2003	2006
**Residence end** **year**	2014	2014	2003	2006	2014
**TCL first name ^a^**	HELENA	MICHAEL	MIZIMALLI		MABINA
**TCL second** **name ^a^**	MSHIMO	MALIGANYA	NDALAHAWA		PALO
**TCL third name ^a^**					
**HH member first** **name**	LUZALIE	JOSEPHI	KOYA		DOTTO
**HH member** **second name**	MATHIAS	BONIFASI	SAHANNI		SALU
**HH member** **third name**					
**True HDSS ID ^b^**	22341597005	77537712004	10012368001	-	10025490004
**True ID in fake** **input dataset**	30	98	1	-	54

Abbreviations: ID - identifier; TCL - ten-cell leader; HH - household; HDSS - health and demographic surveillance system Ten-cell leader: a ten-cell leader is an individual who acts as a leader for a group of ten households and these positions have been relatively stable over time True HDSS ID of patient (found in fake input dataset), which is unknown in reality

### Case 2

The patient enters the ANC and agrees to take part in the study. The fieldworker collects her ANC ID, but also notices she carries an HTC card, so they collect that information as well (these cross-clinic links are common in our fieldwork and allow us to link patient records across multiple services). The fieldworker also enters the personal and residence details she reports ( Table 1). The software displays the top 20 potential matches to the fieldworker. The fieldworker selects the top ranked record to view the entire household membership and confirms the reported co-resident is listed. The years of residence are only off by one year, and the birth year and residence details match exactly. There are minor spelling mistakes in the names reported, but the reported names are switched in order on the HDSS record, which is not uncommon for the data in this setting. The fieldworker assigns the match to this record and ends the search as all reported residency episodes were found. The fieldworker saves a match note that says, “All reported residency episodes found.” The fieldworker then stores the visit date and thanks the patient for her time.

### Case 3

The patient enters the HTC and agrees to take part in the study. The fieldworker collects her HTC ID and enters it into the system along with the personal identifiers she reports ( Table 1). During the interview, she reports she had two residency episodes in different villages, one from 1995 to 2003 and the other from 2006 to 2014. The patient reports to have lived outside of the HDSS area between 2003 and 2006. The fieldworker enters the information for the most recent residency episode and initiates the search. The software displays the top 20 potential matches from the HDSS to the fieldworker. The fieldworker selects the top ranked record to view and confirm that the other household members are correct. There are minor spelling errors in the names and the year of birth is off by one year, but the residence details are the same, so the fieldworker assigns this record as a match.

The fieldworker continues moving down the list of potential matches and tries to find the record associated with the older residency episode. However, the fieldworker finishes going through the list without detecting the record. The fieldworker informs the patient that her record for the older residency episode was not found and asks if there was any reason why her personal details would have been different. She informs the fieldworker she was married in 2003 and provides her maiden name and the name of another household member for that episode. The fieldworker amends the personal details and attempts a second search. The fieldworker now finds the top ranked record to have a few spelling differences, but the years of residence, village, and birth year are all the same. Additionally, the household member is listed on the record. The fieldworker assigns the match to this record and ends the search as all reported residency episodes were found. The fieldworker saves a match note that says, “All reported residency episodes found.” The fieldworker then stores the visit date and thanks the patient for his time.

### Return visits

When any of the case patients return to a linkage clinic, their clinic IDs when entered will retrieve the match status (in this case, “Matched’; if no matches were made, “Not matched”) and the saved match notes. In these cases, the fieldworker can quickly see no other searches are needed and can simply store the new visit date before thanking the patient again for their time. In the event a match note stated, “Missing a record for 2002–2007 in Kisesa Kati,” the fieldworker can focus the interview to obtain the personal details that were associated with that record.

## Conclusions

The PIRL software – which combines a probabilistic search algorithm for identifying potential matches with a relatively simple human intervention – has shown promise for linking multiple data sources without a unique identifier in rural Tanzania. A key advantage of this software over other software that employ purely automated record linkage is the ability to perform multiple searches for the same individual. This is of importance for individuals whose records are more likely to contain out-of-date or inaccurate names or addresses, particularly for individuals with older residency episodes and women whose names change after marriage. Each search attempt on the HDSS database takes less than 15 seconds to complete. Excluding time spent obtaining written consent, the median duration of time we spend with each patient is six minutes.

A limitation of the search database in the current implementation of the software is that it can only be as current as the most recently completed HDSS round. In Kisesa, HDSS rounds are conducted for a few months roughly once per year, and extensive data cleaning delays the data availability by another few months. Therefore, recent residents, such as children and adults who first move into the HDSS area or infants born after the last HDSS round, will not have an HDSS record. The software allows the user to input the date of first residence in the HDSS area, so that these individuals can be flagged in subsequent analyses. During the first 18 months of operations in Kisesa, we flagged 1,576 (24.7%) patients as recent residents out of 6,376 clinic attendees who consented to the linkage study.

In this setting, a purely automated retrospective approach to record linkage would have only correctly identified about half of the true matches and resulted in high linkage errors, therefore highlighting immediate benefit of this prospective approach ^19^. Linking health records to an HDSS database generates a rich data source of directly observed data on access to and utilization of health facility services at a subnational level.

## Data and software availability

Software source code: https://github.com/LSHTM-ALPHAnetwork/PIRL_RecordLinkageSoftware

Archived source code as at time of publication: https://doi.org/10.5281/zenodo.998867 ^23^

License: MIT

Due to ethical clearances, we are unable to share identifiable HDSS data or clinic identifiers used in our implementation of the software with anyone outside the study team. However, demographic data only for the HDSS are available via the INDEPTH Network’s Sharing and Accessing Repository ( iSHARE). Applications to access the anonymised data for collaborative analysis are encouraged and can be made by contacting the project coordinator for the Kisesa HDSS, Mark Urassa ( urassamark@yahoo.co.uk), or by contacting the ALPHA Network team ( alpha@lshtm.ac.uk).

## Supplementary material

**Supplementary File 1.** Kisesa-HDSS record linkage user guide.

**Supplementary File 2.** Fake input dataset with codebook.

**Supplementary File 3.** Script to create fake input dataset.

**Supplementary File 4.** Output datasets for case patients with codebook.
