# Supplementary material for: Point-of-contact Interactive Record Linkage (PIRL): A software tool to prospectively link demographic surveillance and health facility data
Source: Gates Open Res. 2018 Jan 11;1:8. Originally published 2017 Nov 6. [Version 2] doi: 10.12688/gatesopenres.12751.2 (PMC5841575; doi:10.12688/gatesopenres.12751.2)
Supplement: Supplementary file 1 [file gatesopenres-1-13850-s0000.tgz › 479f8ddd-3893-4b6e-8146-e056e438fc63.docx]

PIRL (Point-of-contact interactive record linkage) User Guide

Software v2.5

This user guide is aimed at fieldworkers, data managers, and other technicians who may utilize the detailed record linkage software within to link individual’s records between the Kisesa Health Centre and the Kisesa HDSS

A collaboration between Mwanza NIMR and LSHTM

|  |  |  |
| --- | --- | --- |
|  |  | Date last edited: 24/01/2017 |
|  |  |  |

Contents

[Overview 1](#_Toc467680020)

[Abbreviations 2](#_Toc467680021)

[Setup 3](#_Toc467680022)

[Logging in 4](#_Toc467680023)

[Before you begin linking 5](#_Toc467680024)

[Record linkage process 6](#_Toc467680025)

[Repeat patients 20](#_Toc467680026)

[Logging off 22](#_Toc467680027)

[Removing a record from the database 23](#_Toc467680028)

[Annex 1 – Installing the software 24](#_Toc467680029)

[Annex 2 – Import/export routine 27](#_Toc467680030)

[Contact Information 29](#_Toc467680031)

[Organization Information 29](#_Toc467680032)

# Overview

The Point-of-contact Interactive Record Linkage (PIRL) system is made for the purpose of linking patient information between HIV services offered in Kisesa Health Centre – including care and treatment clinic, HIV testing and counseling clinic, and the antenatal clinic – to the Kisesa demographic sentinel surveillance system (Figure 1).

Figure 1. Graphical representation of record linkage

**
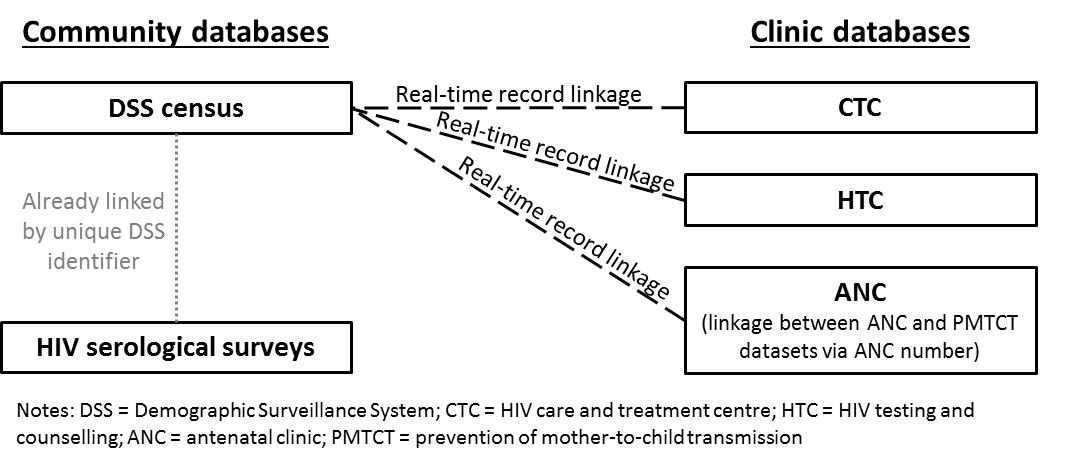
**

# Abbreviations

ANC – antenatal clinic

BMC – Bugando Medical Center

CTC – care and treatment clinic

DSS – demographic sentinel surveillance

HEID – HIV Exposed Infant ID

HTC – HIV testing and counseling clinic

PIRL – Point-of-contact Interactive Record Linkage

PMTCT – Prevention of Mother-To-Child Transmission clinic

TGRF – Tazama Green Referral Form

# Installation and setup

Annex I and Annex II carry instructions on how to install and setup the record linkage software. Before the software will run, all steps in Annex I need to be completed. If you require any importing or exporting of data into or out of the record linkage system, you will also need to complete the steps in Annex II.

If you are working on a machine that already has the record linkage software setup, you can move forward to the next section.

# Logging in

1. Double-click the **TazamaDSSClinicLinker** application file (should be on the computer desktop. If not, located at \\TazamaDSSClinicLinker\bin\Debug\TazamaDSSClinicLinker.exe)
2. A log-in window will appear after a few moments (Figure 2)
3. Select the Server “**.\KISCLINICDB”**
   1. The “.” is only a placeholder for the name of the machine that you’re on, so if you are on machine called “RECLINKA”, the Server will be RECLINKA\KISCLINICDB
4. Select the Database “**KisesaDSSClinicLinkSystem”**
5. Type in the User Name = **Admin**
6. Type in the Password = **@Admin** (*this can be changed in SQL*)
7. Click OK to open the linkage software
   1. If you receive an error, it is usually because you did not type in the correct User Name/Password combination. Please try again.

Figure 2. Blank Login Window Figure 3. Completed Login Window


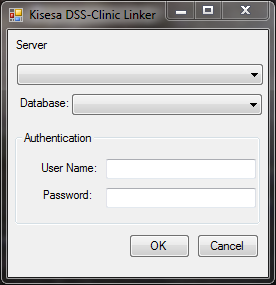

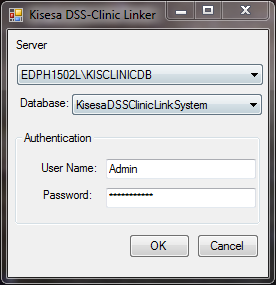


# Before you begin linking

- Select which “Health Facility Department” you are working in (Figure 4)
- The department that you select will stay the same for every session until you close out of the linkage application completely
- If you do move to a different department in the same day, you can simply change the department selection

Figure 4. Select Health Facility Department


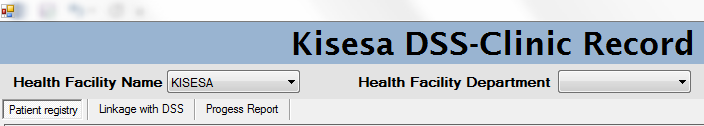


- Options in the Department drop down list are:
- CTC = Care and Treatment Clinic
- HTC = HIV Testing and Counselling clinic
- ANC = Antenatal clinic
- PMTCT = Prevention of Mother-To-Child Transmission clinic
- PMTCT is not a separate building or facility. It is run out of the ANC. You would select PMTCT on the days that the ANC cares for HIV-infected individuals (currently on Wednesdays)

# Record linkage process

- The following diagram (Figure 5) depicts an overview of the process, or flow, of the record linkage sessions. Each of these boxes are described below.

Figure 5. Record Linkage Flow Diagram


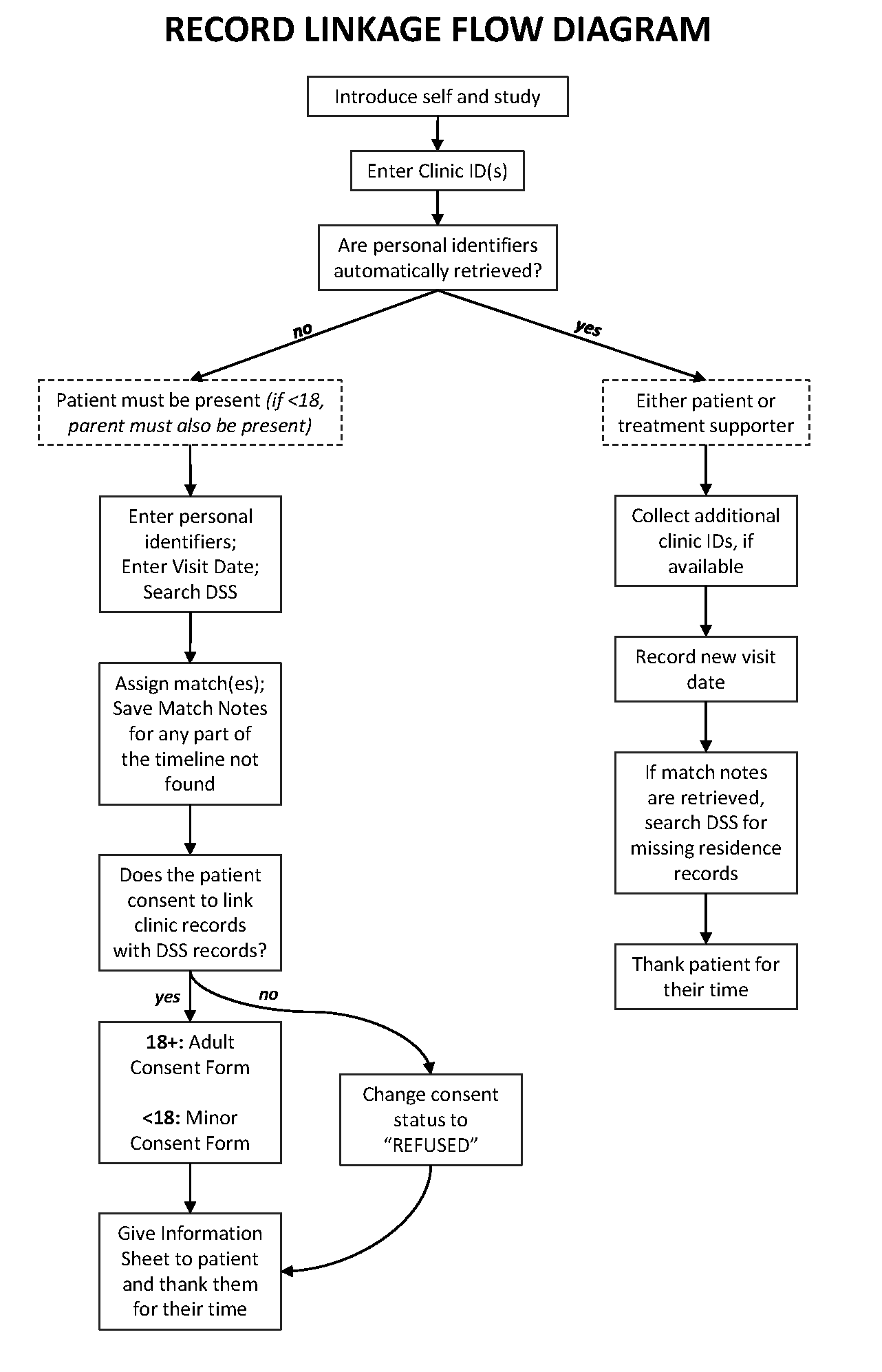


## 1. Introduce self and study

- Before you start asking the patient for their name and residence information, reasonable time should be spent introducing yourself and the record linkage research
- This introductory speech should include the following elements:
- Your name
- Your affiliation with Tazama and NIMR
- An overview of what record linkage is
- What to expect during a record linkage session
- We will ask for name, DOB, residency history from 1994 to current, Balozi name, and household member name
- An assurance that we will not contact the patient or anyone mentioned during the session outside of the clinic
- Sessions for new patients should last 10-15 minutes; return patients about 5-10 minutes
- It is not a requirement to participate in record linkage in order to receive normal clinic services

## 2. Enter Clinic ID(s)

- Each of the clinics assigns various unique IDs depending on age/HIV status (Figure 6).
- Individuals will have IDs assigned to them from any clinic they receive care from. So, if an adult woman attends HTC in 2013 and CTC in 2014, she will have an HTC ID, TGRF, CTC ID, and File Ref.

Figure 6. Unique IDs assigned by clinic and type of patient


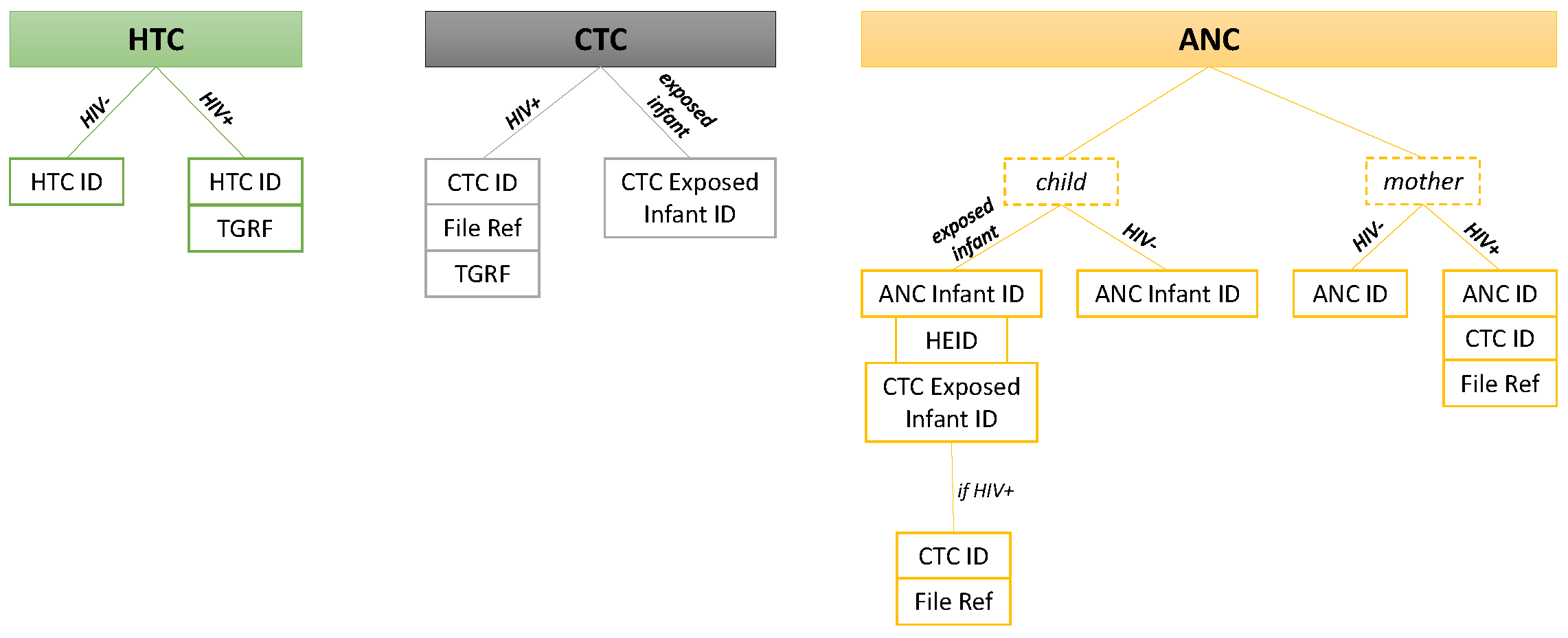


- Once the patient has agreed to take part in record linkage, collect all clinic IDs associated with the patient and enter them into the software (Figure 7).

Figure 7. Clinic ID fields


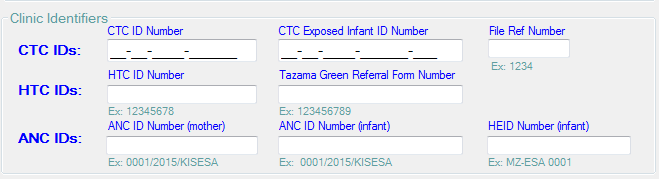


1. CTC ID Number: a unique 14 digit number for each patient receiving care. The current 14 digit mask was preceded by 8 or 11 digit masked CTC IDs, but these old forms are easily transformed to the current 14 digit mask (Figure 8).

Figure 8. CTC ID Conversion Chart


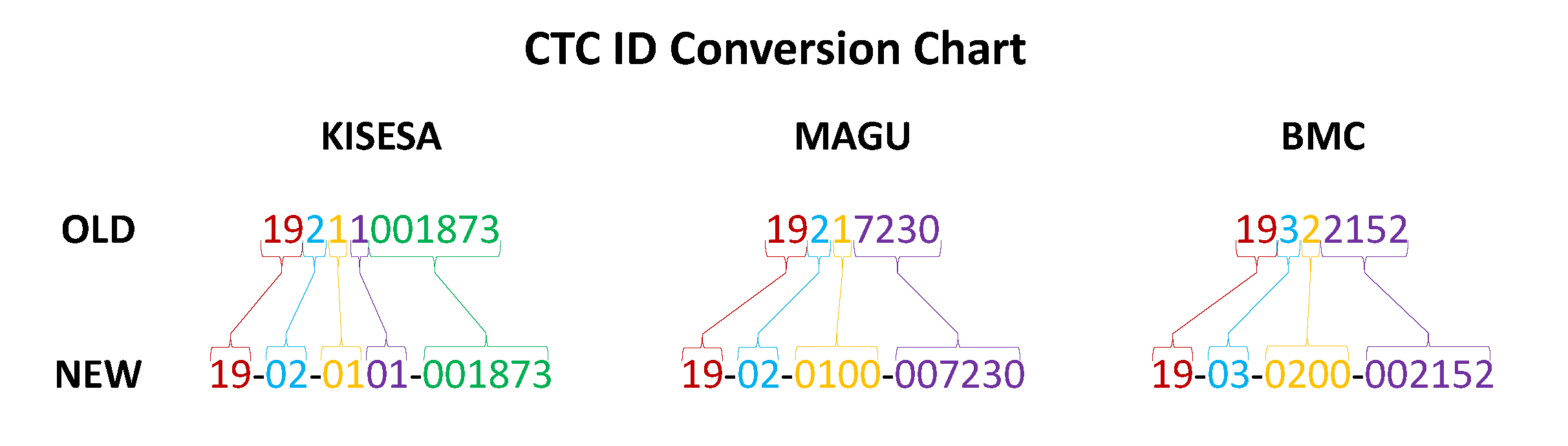


*BMC = Bugando Medical Center

1. CTC Exposed Infant ID Number: this follows the same format of the CTC ID Number, except it ends in “-C##”. The “C” stands for “child” while the “##” is the number of children the HIV-infected mother had during her HIV infection. For example, a mother who has her first child after she was infected, the child receive a CTC Exposed Infant ID Number that is exactly the same as his mother’s CTC ID Number, with the additional “-C01” at the end. Therefore, if the mother’s CTC ID Number was 19-01-0101-123456, all of her children would receive CTC Exposed Infant ID Number’s of 19-01-0101-123456-C01 (1^st^ child), 19-01-0101-123456-C02 (2^nd^ child), 19-01-0101-123456-C03 (3^rd^ child), etc. If after the child has received the Option B+ treatment schedule s(he) is HIV-positive, the child will then receive their own CTC ID Number.
2. File Ref Number: every patient in the CTC should also have a File Ref Number in addition to their CTC ID number. File Ref Numbers are written on the CTC2 return card and are 4 digits in length.
3. HTC ID Number: this is an 8 digit number that is created at NIMR during the production of the HTC logbook and HTC return cards. The number follows a modulo 97 format.
4. Tazama Green Referral Form (TGRF) Number: this number is from the referral forms completed in HTC and taken to CTC when a client is detected HIV-positive in the HTC. This number follows a modulo 97 format.
5. ANC ID Number (mother): the format for this number is serial # in registration/year of registration/village where care was initiated for that pregnancy. The serial number starts at 0001 every calendar year.
6. ANC ID Number (infant): follows the same format as ANC ID Number for the mother.
7. HIV Exposed Infant ID (HEID) Number: this number is just the village name where the birth occurred and a serial number. The format used for a child born to an HIV-infected mother in Kisesa is MZ-ESA ####.

## 3. Are personal identifiers automatically retrieved?

- As you begin collecting all the relevant clinic IDs, if you have already seen this patient in the past, all the personal identifiers will be automatically filled in.
- Similarly, if the patient has been seen in another clinic that conducts record linkage, and you enter a clinic ID from the other clinic, all the personal identifiers will be automatically filled in.
- Only after you have collected *all* relevant clinic IDs, can you determine if the patient is a new patient or a repeat patient.
- If it is a new patient, you will be able to click out of the clinic ID fields and begin typing in the other personal identifiers, such as First Name. If you enter all clinic IDs and the First Name field remains blank, then this is a new patient. Continue to the next section, #4.
- If it is a repeat patient, you will see a message box appear on the screen (Figure 9). If you see this message, skip to section on repeat patients on page 20.

Figure 9. Message box for repeat patients


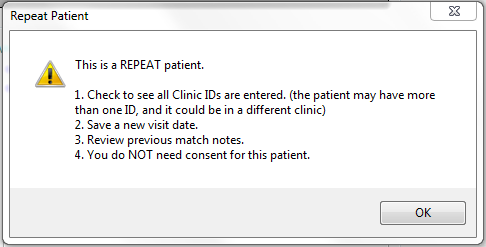


## 4. Enter personal identifiers; Enter Visit Date; Search DSS

- Once you have determined that it is a new patient, first start constructing a residency timeline for the patient (Figure 10). This will be very handy when completing the rest of the linkage process. As you see in the figure below, the DSS data we search covers dates from 1994 to 2014. The goal of this task is to obtain verbally all the residences the patient has had in these years. Helpful questions to ask are, “Where do you live now?, Have you always lived there?, Have you been married, and if so what was your name before and after marriage?” Because we want to find every DSS record each person has, and things like village of residence and names can change over time, it is important to ask for any major life events that would indicate a change in residence or name. Getting this information at this step will be very useful for when you actually are searching the DSS records.

Figure 10. Examples of residency timelines


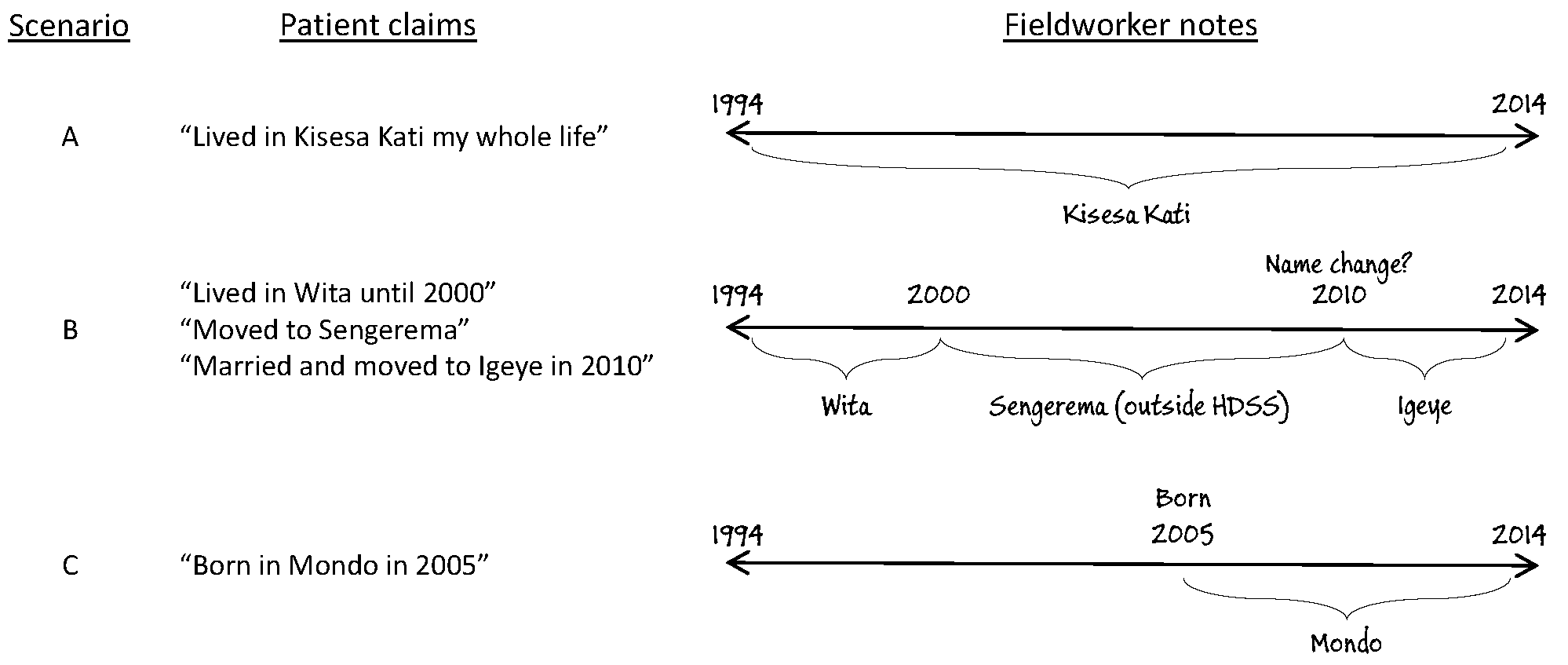


- Once you have finished building a residency timeline for the patient, begin entering the personal identifiers (Figure 11). You will enter a new set of residence details for each residency episode the patient claims to have in the DSS area.

Figure 11. Personal identifiers including residence details


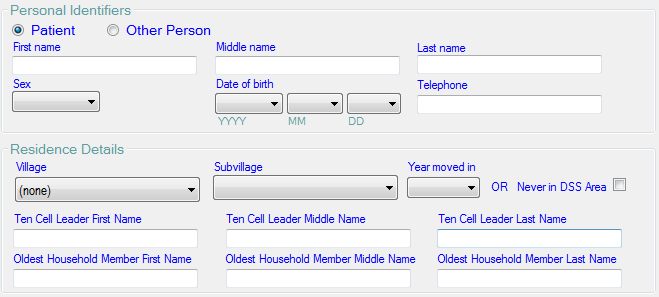


1. Search target (“Patient” or “Other Person”): For new patients you will always begin searching for the individual’s DSS record(s) by using their information. That means that “Patient” should always be selected for at least the first search through the DSS. However, if you are having difficulties locating one or more expected DSS records, you can select “Other Person” and search the DSS for another individual who shared a residence with the patient. An example is searching on the patient’s spouse or parent, if the patient claims to have lived with them and you are having trouble finding that record.
2. Names (First, Middle, and Last): Collect as many names as the patient is willing to give. Because you are attempting to match names as they are listed in the DSS, a good idea is to ask the patient for names that they use in each of the households they claim. We know that many patients in the clinic use different names than they use in their village. So think ahead and ask for other names, such as tribal names, names before and/or after marriage, nicknames. Also, it is good to ask the patient to spell their names for you if you are not absolutely sure of the spelling. All of these tips will help you in matching the names you type in to the software to the names listed in the DSS. At least one name is required and should always be placed in the First Name box. If the patient cannot provide a Middle or Last Name, that is okay.
3. Sex: options are in a dropdown box. Male, Female, Unknown
4. Date of birth: these fields are separated into year (“YYYY”), month (“MM”), and day (“DD”). Most patients will either know or can provide an estimate of their year of birth. It is okay to leave any of these fields blank if the patient does not know.
5. Telephone: collect the patient’s primary SIM numba
6. Village: When you get to this field, you should stop using the software and begin building the timeline in your notebook (Figure 10) if you haven’t done so already. Once you have a completed timeline of residence history between 1994 and the year of the last DSS round, you can then begin entering the residence details into the software. Use the listed residences on your timeline to guide your search for DSS records one residency episode at a time. Remember, do not start by asking patients if they live in Kisesa (this is because Kisesa means something different to each patient and the meaning of Kisesa as a ward has also shifted over time). Always begin collecting information about a patient’s residencies by letting them state the villages they have lived in. If the patient claims to never live in DSS area, then skip to #9.
7. Subvillage: For each village that you select, the available list of subvillages in that village will be available to choose from in this dropdown box. Select the subvillage the patient claims to live/have lived in.
8. Year moved in: this information should also be read from your created timeline (Figure 10). For each residency episode, select the year the patient claims to have moved in to the village/subvillage you selected. It is okay to leave this field blank if the patient does not remember.
9. Never in DSS area checkbox: If the patient claims to never live in any of the villages within the DSS area, select this checkbox and leave Village/Subvillage/Year Moved In fields blank. It is possible, however, that you may find DSS records for patients who claim to have never lived in the DSS area. This is not a problem. Even if the patient claims to have never lived in DSS area, still move forward with searching the DSS database to try and locate records.
10. Ten Cell Leader/Balozi Names (First, Middle, Last). The Balozi list is updated every DSS round. Therefore, all Balozis in our database are the Balozis found at each household in 2014. Ask the patient if he or she knows the current Balozi (which should mostly match the Balozi in 2014) at the household you are searching. If you are searching for a residency that is a current residence, then you simply ask who the current Balozi at the household is. However, if you are searching for a past residency, say one that occurred between 1994 and 2000, the Balozi listed on that record will still be the Balozi at that household in 2014. It is okay to leave any of these fields blank if the patient does not know.
11. Oldest Household Member Names (First, Middle, Last): Ask the patient to give the names of the oldest household member in their household. Similar to Balozi names, there is a time element involved. If you are searching for a current residency, then simply ask who the oldest living household member is now. However, if you are searching for a past residency, say one that occurred between 1994 and 2000, the household member should be the oldest living household member when they exited that household, so in 2000 in this example.

- After you entered the personal identifier and residence details, click “Save for Search” immediately below where you entered the residence details (Figure 12).

Figure 12. Save, edit, and end buttons on the Patient Registry Page


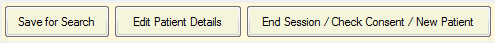


- After you clicked “Save for Search”, enter the visit date information at the top right portion of the Patient Registry page (Figure 13).

Figure 13. Visit information


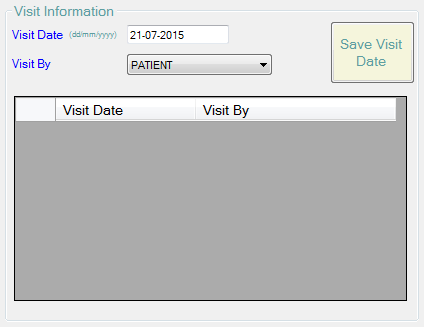


- The date of the visit is automatically filled for you to the current date
- The “Visit By” dropdown box is automatically filled to “PATIENT”, as almost all visits will be made by patients themselves. However, in the CTC only, repeat visits are able to be done by a “TREATMENT SUPPORTER”. If a treatment supporter is seen in the CTC, and the software recognizes the clinic IDs as a repeat patient, then it is okay to save new visit information as long as you select “TREATMENT SUPPORTER” from this dropdown box.
- For most patients, you will not need to change anything in these fields, and you can simply click “Save Visit Date” (Figure 13).
- Now you are ready to begin searching the DSS database for matches!
- Click on the page tab called “Linkage with DSS” found at the top left of the screen underneath the Health Facility Name (Figure 14).

Figure 14. Page tabs


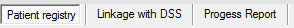


- On the Linkage with DSS page, click “Search DSS” (Figure 15).

Figure 15. "Search DSS" and "Assign match" buttons


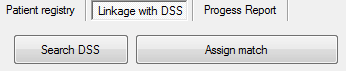


- BE PATIENT! Currently, the software application takes 10-20 seconds to return its search results. Do not begin clicking on other things in the software while it is searching. Take this time to make sure you have a completed timeline (Figure 10).

## 5. Assign match(es); Save Match Notes

- When the software completes its search, the top 20 likely matches will be displayed on the bottom half of the screen, in order of how similar the record matches your inputs (Figure 16).
- Beginning with the top record, go through each record and determine whether that record pertains to the individual who is sitting in front of you.
- When you select each record, the entire household members list will appear in the bottom right corner of the software window.
- For each record, look through the household members list to see if the “Oldest Household Member” that the patient gave to you is in the list.
- If the name is in the list, this record is likely to be the patient’s record. However, you may have found a record for someone else who lives in the same household but looks similar to the patient, in terms of similarity of names, date of birth, and/or gender. Therefore, it is still important to make sure the record is for the patient by looking at all the available information in the record (name, date of birth, date of residency) to determine if the record is a true match to the patient you are interviewing.
- If the patient did not give you a name or if the name is not in the list, it is important to still ask the patient whether they know any of the listed household members for each record. In general, you can get a sense of whether the record is a likely true match if the patient says they know and/or lived with a few household members in the list.

Figure 16. Search results (blank for security purposes)


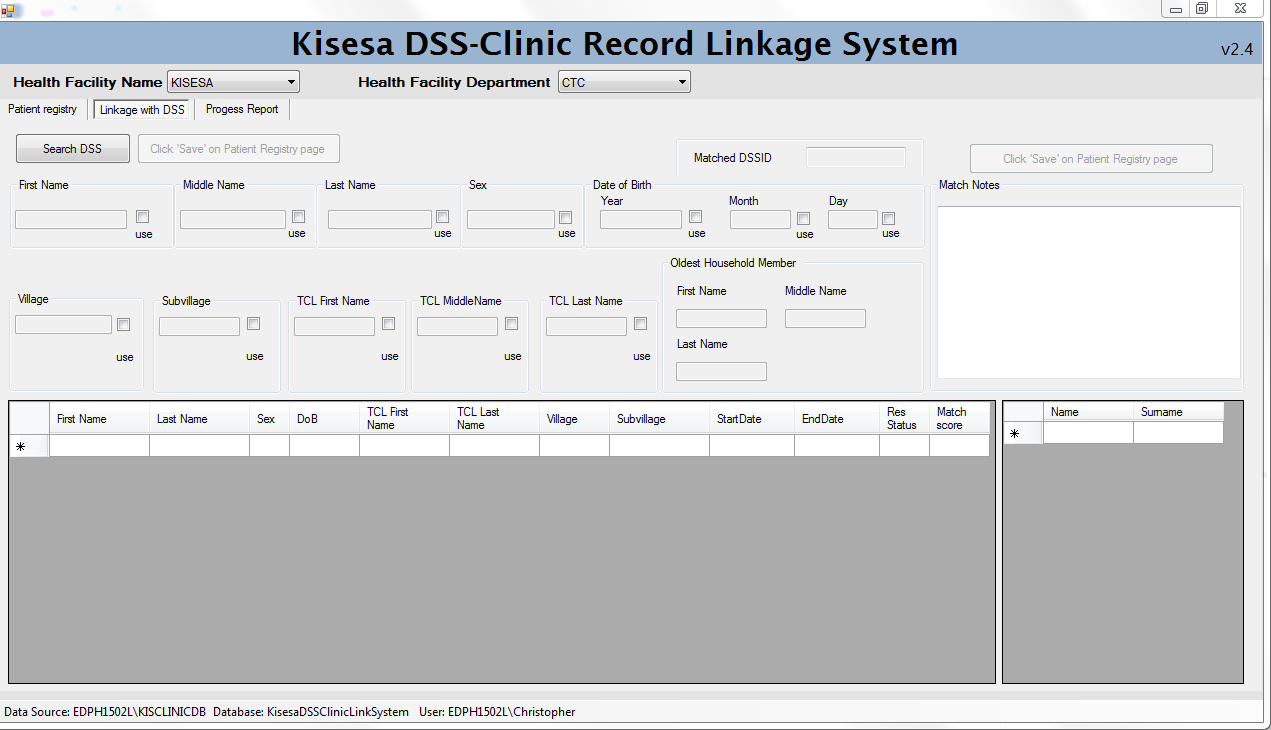


- It is important to know that not all parts of the found DSS record may match up with what you typed into the system. This is generally okay! We are searching for records that potentially go back to 1994, and many things can change over that time period. For example, people may get married (and have a name change), or people may move (and have multiple records in a variety of village/subvillages).
- It is important to remember that we are search for records in the DSS database. That is, the information that the patient gave to the DSS interviewer at their home residence is what we should be searching for. There are times when patients may use a different name in the clinics where we meet them than they use at their home. Additionally, they may have never spoken to a DSS interviewer because usually only the head of household gives all information (e.g., name, date of birth) to the DSS interviewer. In these cases, the name that is stored in the DSS database is the name the head of household used for them, and that may not match up with what the patient uses for themselves outside of their home. If the patient claims to be from the Kisesa DSS area and you have difficulty finding any DSS records for them, try asking about other names they use at home or elsewhere. It is most important to remind the patient that we will not ever contact the patient outside of the clinic.
- Because our DSS database that we search is only updated annually, there are patients that we will interview that claim to live in the DSS area (and they do!), but they moved in after the last DSS round and/or our search database has not been updated yet. This is unfortunate but is the way of how we update the DSS database. If this happens, you will know when the patient tells you they only moved into the DSS area recently and had no previous residence history in the DSS area. Still, continue by searching for this patient in the DSS database (as we do with all patients regardless of what they tell us) just in case there is a record of them in the DSS database. This happens when the head of a household tells the DSS interviewer that the patient lived in that household, even though the patient did not consider themselves to live at the residence. It is not our job to inquire about the specific and sometimes unique living arrangements of the patient – our only goal is to see if we can find a record for them in the DSS database.
- This is a particular issue in the ANC as many pregnant women will move back home or closer to the clinic when they need ANC services. That means that they will have moved into Kisesa DSS area within the last 9 months, and our DSS databases wouldn’t have been updated to capture their residence yet.
- Once you have found a match, highlight the record and then select “Assign match” (Figure 17)

Figure 17. Assign match button


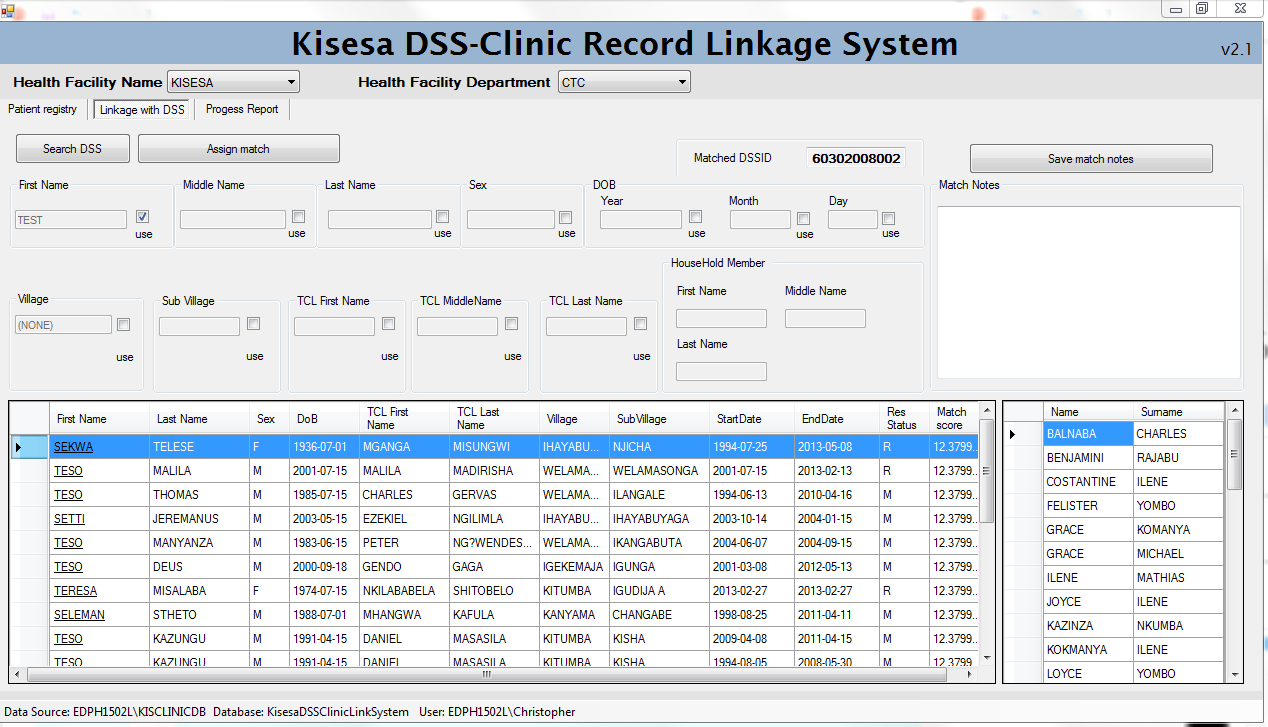


- Because we are searching for every residence a patient lived in back to 1994, some patients will have more than one DSS record if they moved within the DSS area. You can assign matches to more than one DSS record if they are all true matches to the patient. As you move from record to record on the top 20 likely matches list, make sure you are making notes on your residency timeline you have constructed for that patient. This will help you understand which time periods you have not yet found.
- If at any point during the search you would like to change some of the information you used to search on, you can go back to the “Patient registry” tab, click “Edit Patient Details” at the bottom of the page, and then add/remove/edit information. Once you are ready to search again (e.g., using a different name, residence details), you can go back to the “Linkage with DSS” tab and click “Search DSS” again. It will take 10-20 seconds each time you click “Search DSS” to update the results at the bottom of the screen. Once they have been updated, you can begin clicking on the top record and continuing the search for the true DSS records.
- If you have found all matches (according to your residency timeline), you can then go back to the “Patient registry” tab and select “End Session / Check Consent / New Patient” (Figure 12). Then you can proceed to the next section “6. Written Informed Consent” on page 18
- However, if you have not found one or more DSS records that you expect to find, and you have exhausted all interview techniques (e.g., asking for other names, etc), then you can save all useful information the patient has offered into the Match Notes box (Figure 18).

Figure 18. Match notes


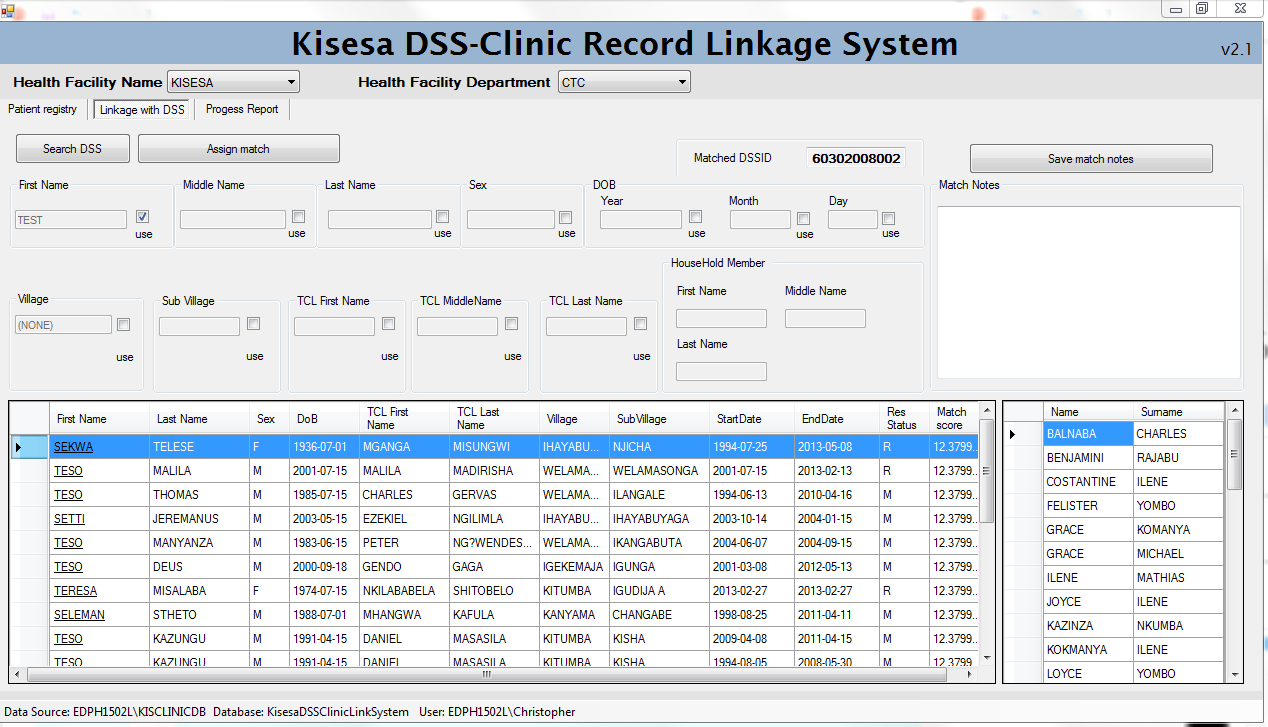


- All information you type in the box will be retrieved the next time you or another fieldworker sees this patient, so be as helpful as possible by thinking of what information you would want to read if you were the one to see the patient next.
- Always put a date for the match note you are saving. Over time, as you see the same patient repeatedly, you will be able to see the entire history of the patient’s match notes. This will help you review what previously was done in terms of searching for DSS records, and this will help you focus your current search on the missing records yet to be found.
- The information you save as a match note should help you re-construct the patient’s missing parts of their residency timeline without having to ask any questions. For example, if you found all DSS records for a patient except for one when he lived in Kisesa Kati from 2000-2002, then an example match note would be “21/11/2016 did not find record for Kisesa Kati from 2000 to 2002”.
- Also write information in the match notes box that could help another fieldworker focus their questions on other reasons why the record wasn’t found. For example, if you asked the patient if they have ever been married or went by a different name, make sure to write that information in the match notes box because that will help the next fieldworker know that the patient has already been asked this question. The fieldworker can then try to think of other reasons why they are missing a DSS record.
- Lastly, you should write any information in the match notes that *explains why* there was a missing DSS record(s). We are very interested in knowing why we are unable to find DSS records for patients who claim to have lived in the DSS area. One reason could be that they live in a small household (perhaps alone or with one other person) and the DSS interviewers did not find anyone at the patient’s home when they went to collect data.

## 6. Written Informed Consent

- When you have finished your search with a patient, click on “End Session / Check Consent / New Patient” on the “Patient registry” tab.
- The software automatically detects whether you have seen this patient before or not. If you or any other fieldworker has not seen the patient before to get consent, a notification will appear to instruct you to offer written consent (Figure 19).

Figure 19. Consent needed alert


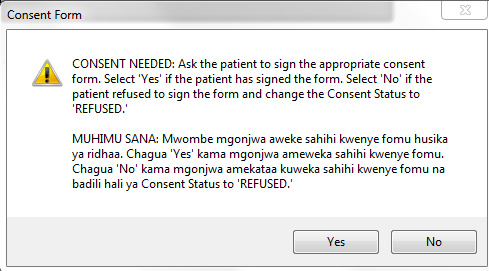


- If the “Consent needed” alert box appears, go through the steps of informed written consent. If the patient is 18 or older, offer the Adult Consent Form. If the patient is 17 or younger, off the Child Consent Form. Once the patient signs the consent form, select “Yes” in the alert box.
- If the patient wishes not to give written consent to be a part of the study, select “No” on the “Consent needed” alert, and change the Consent Status box to “REFUSED” (Figure 20). Once the data is collected and merged for the day, all data for patients who refused consent will be deleted permanently.

Figure 20. Consent refused


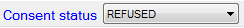


- If, when you select “End Session / Check Consent / New Patient”, no “Consent needed” alert box appears, then you do not have to offer informed written consent because it has already been obtained on a previous visit.

## 7. Patient Information Sheet

- After every patient visit, offer every patient (whether they consented or not) a Patient Information Sheet. This contains details on the study as well as contact information in case any question arises after they visit with the fieldworker.

# Repeat patients

## 1. Collect additional clinic IDs, if available

- As you begin collecting all the relevant clinic IDs, if you have already seen this patient in the past, all the personal identifiers will be automatically filled in.
- If it is a repeat patient, you will see a message box appear on the screen (Figure 9).
- As shown in Figure 6, it is possible for a patient to have multiple clinic IDs – not only within one clinic, but across many clinics. Therefore, it is important to collect as many clinic IDs from each patient at every visit.

## 2. Record new visit date

- Once you have ensured you collected all clinic IDs a patient has, carry on to record a new visit date, as outlined in Figure 13 on page 13.

## 3. Check match notes

- Finally, it is possible that not all DSS records were found for the patient at their last visit. If the patient has previously been matched to at least one DSS record, the text “MATCHED” will appear in the Match Status field (Figure 21). If the patient has not been matched to any DSS record, the text “NOT MATCHED” will appear in the Match Status field.

Figure 21. Match Status


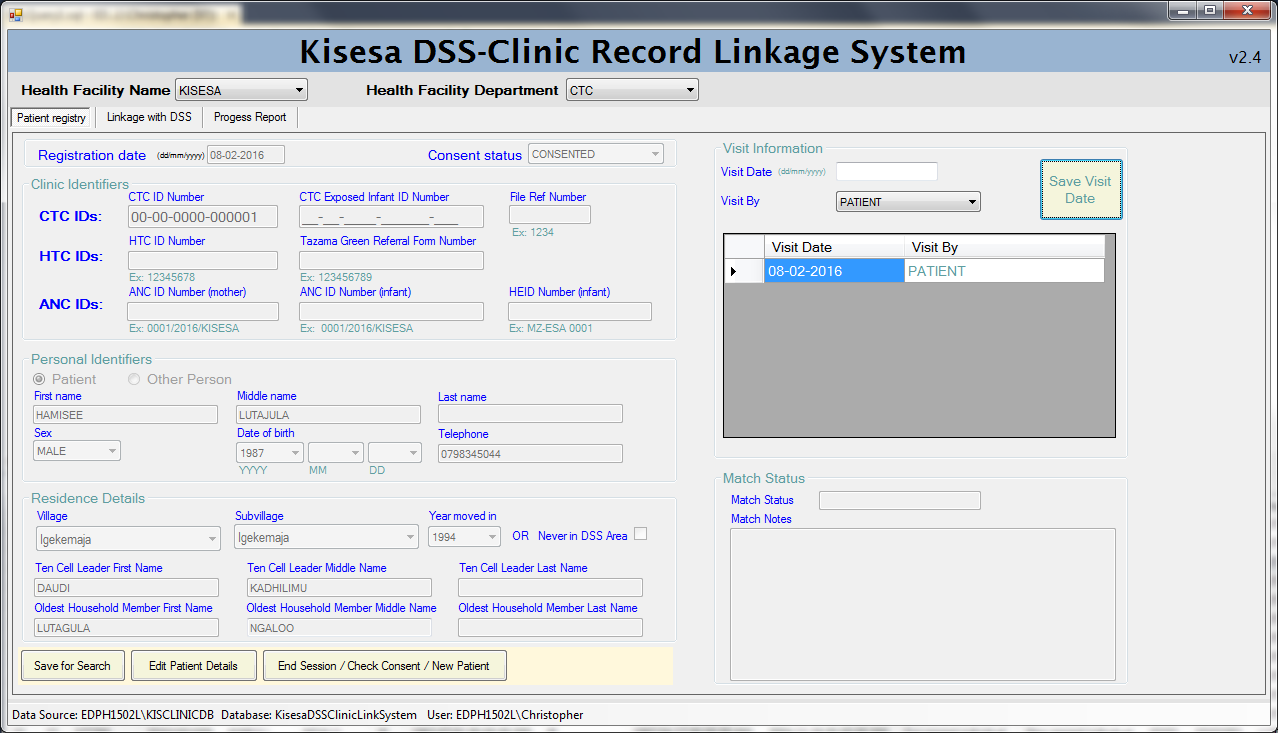


- Regardless of whether the Match Status says “MATCHED” or “NOT MATCHED”, always refer to the “Match Notes” test box to read details about the patient’s previous visits. If any or all DSS records were not found in a previous visit, proceed with performing another search for the patient’s missing DSS records.
- If you still have issues with finding any DSS record, or you were given any additional information why the patient may be missing from the DSS database, write and save those notes in the Match Notes text box in the “Search for DSS” tab (Figure 18).

# Logging off

Whenever you have finished performing record linkage and are ready to shut down the computer, first make sure that there are no patient details displayed on the “Patient Registry” tab. If there are details, simply click the “End Session / Check Consent / New Patient” button. After that, you can close out of the software by clicking “X” in the top right hand corner of the window.

# Removing a record from the database

If any patient who has been previously interviewed and linked expresses that they do not want their medical records linked with their community data, the fieldworker will ask the patient for their unique clinic identifying code and pass this information along to the data manager. The data manager can use SQL commands to retrieve the individual’s linked information and delete the link between the clinical and demographic records.

# Annex 1 – Installing the software

1. Download Microsoft Visual Studio 2013 Community edition
   1. IT IS REQUIRED THAT THIS BE INSTALLED FIRST PRIOR TO MICROSOFT SQL SERVER
   2. <https://www.visualstudio.com/products/visual-studio-community-vs>
   3. You will need to either sign in or create a login to download
   4. Once you have the executable installation file “vs_community”, run it to install
   5. Computer will force a restart
2. Download Microsoft SQL Server 2012 Express
   1. <http://www.microsoft.com/en-us/download/details.aspx?id=29062>
   2. It will prompt which download files you want
      1. SQLEXPRADV_x64_ENU.exe
      2. It is 1.3GB, so will take some time to download
   3. Once finished, double click on this executable installation file
   4. SQL Server Installation Center window pops up
   5. Click Installation on left
   6. Click “New SQL Server stand-alone installation or add features to an existing installation” on right
   7. A window pops up
      1. Click the check next to “I accept the license terms.”
      2. Click “Next”
      3. Give it a minute
   8. A new window pops up
      1. Ensure all Features are selected, then “Next”
      2. During Instance Configuration, select “Named instance”, then name the instance “KISCLINICDB”, (instance ID will update once you click in that box) then “Next”
      3. “Next” on Server Configuration (don’t change anything here)
      4. “Next” on Database Engine Configuration
         1. (We will manual change authentication mode later. Leave it as “Windows Authentication” for now)
      5. “Next” on Reporting Services Configuration
      6. “Next” on Error Reporting
      7. Again, it will take a while to download. Be patient.
      8. Says installation was completed successfully
      9. Close out of all installation windows still on screen
3. Open Microsoft SQL Server Management studio (Start>Programs>Microsoft SQL Server 2012>SQL Server Management Studio)
   1. If a window asks you to import customized user setting from a previous SSMS, select “no”
   2. In the Server line, select the KISCLINICDB instance from the drop-down menu
   3. Authentication should remain Windows Authentication
   4. Click Connect
   5. Run S0 sql script to create KisesaDSS database
   6. Run the CreateTableForFakeData.sql script next. If you are uploading your own data, edit this script to your liking.
   7. In the Object Explorer, right click Databases, then click Refresh
   8. Click on + sign next to Databases to drop down list of databases
   9. Using your favorite method, upload the fake data (called Fake100) or your own data into the KisesaDSS database in SQL. We have provided 3 versions of the Fake100 database (.csv, .sas7bdat, and .dat). We use an ODBC connection from Stata to upload the data in.
   10. Once imported, to test if this worked, in the Object Explorer, click the drop down + signs next to Databases, then KisesaDSS, then Tables
   11. You should see a table called dbo.Fake100
       1. Right click this table and select “Select top 1000 rows”
       2. You should see DSS records ordered by idlong variable
   12. Run the S1-S4 SQL scripts and ensure there are no errors
       1. If a window pops up asking you to normalize line endings, Then select “Windows (CR LF)” and click Yes
   13. We need to make sure the security authentication mode is set up correctly
       1. In SQL Server Management Studio Object Explorer, right-click the server, and then click Properties.
          1. On the Security page, under Server authentication, select “SQL Server and Windows Authentication mode”, and then click OK.
          2. In the SQL Server Management Studio dialog box, click OK to acknowledge the requirement to restart SQL Server.
          3. In Object Explorer, right-click your server, and then click Restart.
       2. In the Object Explorer, expand Security, expand Logins, right-click sa, and then click Properties
          1. On the Status page, in the Login section, click Enabled, and then click OK
   14. Exit out of SQL Server Management studio
4. Open the computer’s Control Panel
   1. Click on Administrative Tools
   2. Double-click on Services
   3. Ensure that the “Extended” tab is selected at the bottom of the Services window
   4. Scroll down the list to find the following names:
      1. SQL Full-text Filter Daemon Launcher (KISCLINICDB)
      2. SQL Server (KISCLINICDB)
      3. SQL Server Agent (KISCLINICDB)
      4. SQL Server Browser
      5. SQL Server VSS Writer (*if available*)
      6. SSDP Discovery (*if available*)
   5. Ensure that all five of these have a Status = “Started”
   6. If any of them do not have Status = “Started”, then do
      1. Right-click on the name
      2. Select Properties
      3. In the General tab, change Startup type to “Automatic”
      4. Click “Apply”
      5. Click “Start”
      6. Click “OK” to exit the window
      7. Repeat these steps until all names listed above have a Status = “Started”
   7. Restart computer
5. Paste a copy of the “TazamaDSSClinicLinker.exe” from the .\TazamaDSSClinicLinkerUpgrade\TazamaDSSClinicLinker\bin\Debug folder on the new machine’s desktop
   - 1. Double-click this new icon on the machine to open the linkage software
     2. HAPPY LINKING!

# Annex 2 – Import/export routine

1. Import/export routine setup (ONE TIME ONLY)
   1. Create 2 new folder in C:\ on each computer:
      1. LinkSystemExport
      2. LinkSystemImport
   2. Control Panel>User Accounts>Manage Other Accounts
      1. Create a new account called DatabaseServer with a @Admin password as Administrator
   3. Log into DatabaseServer profile
   4. Open SQL Server Configuration Manager
      1. Double click SQL Server Services
      2. Double click SQL Server (KISCLINICDB)
      3. Change Account Name to .\DatabaseServer
      4. Change password @Admin
      5. Click Apply
      6. Click Restart
      7. Click OK
   5. NOTE: Every program runs as a user. The DatabaseServer login provides the Microsoft database server program, the SQL server service, a user to impersonate as its running. But that's all behind the scenes. Once this setup above has been complete, no one ever has to log into it again – not even data admin unless in the rarest of occasions where you change the software entirely.
2. After each day, ready to sync machines on regular windows profile:
   1. On each employee machine:
      1. Open up SQL Server MS
      2. Run ExportScript_bak.sql
      3. The .bak file is now in C:\LinkSystemExport
   2. On Admin computer
      1. Copy all files into C:\LinkSystemImport
      2. open SSMS
      3. run ImportScript.sql
      4. run S5
         1. see how many records were already on machine
      5. Once datasets are checked and think they are good (whether there was changes or not)
         1. Run ExportScript_bak.sql
   3. On each machine:
      1. Copy on the compiled .bak file from Admin computer into the C:\LinkSystemImport for each employee machine
         1. And run ImportScript_bak.sql
         2. run S5 to make sure every machine matches
