# Supplementary material for: Point-of-contact Interactive Record Linkage (PIRL): A software tool to prospectively link demographic surveillance and health facility data
Source: Gates Open Res. 2018 Jan 11;1:8. Originally published 2017 Nov 6. [Version 2] doi: 10.12688/gatesopenres.12751.2 (PMC5841575; doi:10.12688/gatesopenres.12751.2)
Supplement: Peer review history

# Peer review: Referee response for version 1

**DOI:** 10.21956/gatesopenres.13811.r26151

**Published:** 2017-12-27

**Authors:** Hye-Chung Kum

**Affiliations:** 1 Department of Health Policy & Management, Texas A & M University, College Station, TX, USA; 2 Department of Computer Science and Engineering, Texas A & M University, College Station, TX, USA

This paper is a case study of using an interactive record linkage software at point of contact in Tanzania. Interactive record linkage at point of contact has benefits over retrospective linkages, so makes sense to do this when possible. 

 The paper has some good points, but below are some suggestions for improvement.

 * [CRITICAL] The following sentence is incorrect and should be edited

     - "Deterministic record linkage is a rule-based approach that requires exact matching between one or more identifiers existing in all data sources. However, when common unique identifiers are not available, probabilistic methods can be employed to assign weights based on the (dis)similarity of components (e.g., name, sex, and date of birth) between records."

     - Deterministic RL does not require exact match between identifiers. For example, same soundex of the name is not an exact match but rather an approximate match and can be used in determistics methods. And determinististic methods can be an effective method to link data when common unique identifiers are not available. There are pros & cons to both the determinististic and probablistic approach. A more relevent distinction is between exact match and approximate match. There is a section in the paper about "Agreement conditions vary for each of the parameters." which discuss the degree of approximate match, either as a field or a full record. Calling exact match based algorithms determinististic match is a common but confusing nomenclature. Both determistics and probablistic match can be based on exact match on fields, or approximate match on fields. Due to many issues in real data, approximate matched based algorithms (both determinististic and probabilistic) do better. It is important to not confuse exact match with determinististic methods for this reason. The quality of matching results are comparable for both deterministic and probabilistic methods as long as the process for linkage is well developed (Antonie 2014, Zhu 2015). More importantly, data standardization, cleaning, flexibility on approximate matches are important in both approaches. 

 Antonie L, Inwood K, Lizotte DJ, Andrew Ross J. Tracking people over time in 19th century Canada for longitudinal analysis. Mach Learn. 2014;95(1):129-146. doi:10.1007/s10994-013-5421-0.

 Zhu Y, Matsuyama Y, Ohashi Y, Setoguchi S. When to conduct probabilistic linkage vs. deterministic linkage? A simulation study. J Biomed Inform. 2015;56:80-86. doi:10.1016/j.jbi.2015.05.012.

 * [IMPORTANT] An explanation of the role of household as a unit, as well as how it relates to the linkage task would make the paper more clear. My read of the paper, the record linkage task is for people, yet there is many mention of the word household and it seems there is some important aspect of the household used in the linkage process but not described anywhere in the paper.

     

 * [IMPORTANT] This might be related to the above point. A better description of the EXACT linkage goal would improve the paper. The following are some sections that need better clarification.

     - "One major weakness of the Kisesa HDSS is the lack of reconciling records of individuals who move households within the HDSS area. Therefore, some individuals may have multiple HDSS IDs if they resided in more than one household in the HDSS area since the start of the HDSS in 1994."

         > what does it mean to move households? Do you mean a the composition of the household changes? For example, a daughter from household A, marries and moves to a different household as a wife? Or is this a simple family moved to a new location? What is a household in this context? Is HDSS IDs a person level identifier, that is if there are multiple IDs per person, are these duplicate records that need to be cleaned out of the database? If not, what is the unit of the HDSS IDs? Are those household person IDs (meaning, when a person is in a different household, they should have another ID, even if it is the same person) ?

         

     - The RL process diagram and other explanations in appendix 1 should be better summerized to be included in the main text, as it is important that the reader understand this process, and the paper should be understandable without having to fully read the appendix.

     

     - The real time RL occurs when a patient visits a clinic. Thus, is the goal to identify the correct record for the patient in the HDSS at the time of visit? (which sounds like correct record retrival task). Or is the goal to clean the HDSS of duplicate records at the point of patient visit? Strictly speaking this is a deduplication task, and identifying the duplicate records in only the first step. How to 'clean' the database after identification is more important but not discuss much in this paper. Or maybe it is to identify ALL records relating to the patient in the HDSS at point of visit, and link these records within the HDSS system, leaving the duplicate records along. If this is correct, what id the unit of HDSS ID and why do you need it smaller than a person and keep duplicate records per person. 

         

 * The backup process description could be more clear. Again, the goal of backup is unclear in the paper. Is the goal to consolidate records from all computers in a local clinic then have the local databases synced to the master HDSS database on the cloud once a day?

 * Given the sensitive nature of HIV, a brief discussion on the issue of privacy and what the patient can and cannot see during the process would be good to include in the main paper. Maybe a discussion of future work to improve privacy.

 * Although this paper is about interactive RL, there is no review of the literature on interactive RL. A discussion of the general pros and cons of interactive record linkage along with references would frame the paper better. A focus on the role of the person in the process, what the person needs from the automatic process to do a good job, and how the software meets those need might work well. Below are some references that might help you get started

 Martha Bailey, Connor Cole, Morgan Henderson, and Catherine Massey. 2017. How Well Do Automated Linking Methods Perform in Historical Samples? Evidence from New Ground Truth. Technical Report. Working Paper.

 Gordon Darroch. 2002. Semi-Automated Record Linkage with Surname Samples: a Regional Study of

 Case LawLinkage, Ontario 1861–1871. History and Computing 14, 1-2 (2002), 153–183.

 Hyunmo Kang, Lise Getoor, Ben Shneiderman, Mustafa Bilgic, and Louis Licamele. 2008. Interactive entity resolution in relational data: A visual analytic tool and its evaluation. IEEE transactions on visualization and computer graphics 14, 5 (2008), 999–1014.

 Hye-Chung Kum, Ashok Krishnamurthy, Ashwin Machanavajjhala, Michael K Reiter, and Stanley Ahalt. 2014b. Privacy preserving interactive record linkage (PPIRL). Journal of the American Medical Informatics Association 21, 2 (2014), 212–220.

 Eric Ragan, Hye-Chung Kum, et al. 2018. Balancing Privacy and Information Disclosure in Interactive Record Linkage with Visual Masking. ACM SIGCHI 2018.

 Qiaomu Shen, Tongshuang Wu, Haiyan Yang, Yanhong Wu, Huamin Qu, and Weiwei Cui. 2017. NameClarifier: a visual analytics system for author name disambiguation. IEEE transactions on visualization and computer graphics 23, 1 (2017), 141–150.

I have read this submission. I believe that I have an appropriate level of expertise to confirm that it is of an acceptable scientific standard.

# Peer review: Referee response for version 1

**DOI:** 10.21956/gatesopenres.13811.r26086

**Published:** 2017-11-08

**Authors:** Duncan Smith

**Affiliations:** 1 School of Social Sciences, University of Manchester, Manchester, UK

The paper describes a record linkage application that has been used to link patient records in Tanzania. The difference between this and other linkage applications is that new records are entered with the patient present and therefore able to assist in identifying correct matches. It is only the current patient's new record that is linked against the other records in the database.

 The paper is generally well written. The use cases are useful for illustrating how the system is used in practice. But I do have a few questions / suggestions.

- Where was Reference 17 (Rentsch CT, Reniers G, Kabudula C, et al.) published? The reference is incomplete.
- “The higher the ratio *m _i_* / *u _i_* , the more useful a field is for matching purposes.”
  I wouldn’t put it like that. A very low ratio is also very useful. (The sentence is probably superfluous anyway.)
- “A Jaro-Winkler score ≥ 0.8 was considered a match.”
  Perhaps re-word to make it clear this means a match on the field rather than on the record pair. It is not entirely clear from the description how names are handled. Is a match on name declared if at least one of the Jaro-Winkler scores are ≥ 0.8, or something else?
  Maybe the above sentence could be something like “One or more Jaro-Winkler scores ≥ 0.8 was considered a match on name.”
- The paper explains that the *m _i_* are derived from a pilot study. But where do the *u _i_* come from? They (and the *m _i_*) could be estimated from the database itself. Have the authors considered this?
- “The software automatically detects when a patient has been seen during a previous clinic visit and displays the match status (e.g., matched, not matched) to the user. The dates of all follow-up visits are automatically logged into the system.”
  How does this happen? Automatically suggests without input from the individual. What match status? Automatically logged? (This is explained in more detail later, but it is not clear at this point in the paper.)
- .bak is commonly used as a file extension for backups of arbitrary file types, so what is “.bak format”?
- “The use of nick-names and interchangeable name order (exemplified in Case 2) is accounted for in the linkage algorithm by allowing all pairwise comparisons between reported names and names found in the HDSS data source.”
  There is no need to say this twice in a short paper.
- The software is released under the MIT licence and made available via GiHub. This is a good thing. However, it does use proprietary technologies that might limit its applicability.
- “The software has been developed for machines running a Windows 7 operating system.”
  Does it run on other Windows versions? What are the options (if any) for potential users without available Windows machines?
  (I won't comment on the code here as it's probably not particularly relevant to the paper itself.)
- I think a little more could be added on the privacy aspect. How much of the data in the potential matches is the patient allowed to see / know? Potential matches could easily relate to people who live near the patient or are close relatives. It seems that great care would need to be taken to avoid revealing the identity of others in the database.

I have read this submission. I believe that I have an appropriate level of expertise to confirm that it is of an acceptable scientific standard.
